# Time resolved transient circular dichroism spectroscopy using synchrotron natural polarization

**DOI:** 10.1063/1.5120346

**Published:** 2019-10-31

**Authors:** François Auvray, David Dennetiere, Alexandre Giuliani, Frédéric Jamme, Frank Wien, Bastien Nay, Séverine Zirah, François Polack, Claude Menneglier, Bruno Lagarde, Jonathan D. Hirst, Matthieu Réfrégiers

**Affiliations:** 1 School of Chemistry, University of Nottingham, University Park, Nottingham NG7 2RD, United Kingdom; 2 Synchrotron SOLEIL, L'Orme des Merisiers, Gif-sur-Yvette 91192, France; 3 UAR 1008 CEPIA, INRA, Nantes 44316, France; 4 Laboratoire de Synthèse Organique, Ecole Polytechnique, ENSTA-ParisTech, CNRS, Institut Polytechnique de Paris, Route de Saclay, 91128 Palaiseau, France; 5 Unité Molécules de Communication et Adaptation des Microorganismes (MCAM), Muséum national d'Histoire naturelle, CNRS, Paris 75005, France; 6 Brain Physiology Lab - CNRS UMR 8118, Université Paris Descartes, 45 Rue des Saints Pères, Paris 75270 Cedex 06, France

## Abstract

Ultraviolet (UV) synchrotron radiation circular dichroism (SRCD) spectroscopy has made an important contribution to the determination and understanding of the structure of bio-molecules. In this paper, we report an innovative approach that we term time-resolved SRCD (tr-SRCD), which overcomes the limitations of current broadband UV SRCD setups. This technique allows accessing ultrafast time scales (down to nanoseconds), previously measurable only by other methods, such as infrared (IR), nuclear magnetic resonance (NMR), fluorescence and absorbance spectroscopies, and small angle X-ray scattering (SAXS). The tr-SRCD setup takes advantage of the natural polarization of the synchrotron radiation emitted by a bending magnet to record broadband UV CD faster than any current SRCD setup, improving the acquisition speed from 10 mHz to 130 Hz and the accessible temporal resolution by several orders of magnitude. We illustrate the new approach by following the isomer concentration changes of an azopeptide after a photoisomerization. This breakthrough in SRCD spectroscopy opens up a wide range of potential applications to the detailed characterization of biological processes, such as protein folding and protein-ligand binding.

## INTRODUCTION

Circular dichroism (CD) is an optical property of molecules having chiral structure(s) and/or spatially oriented arrays of chromophores. It manifests itself as a difference in absorption for left- and right-circularly polarized light. In the ultraviolet (UV) range, this feature has been exploited for decades for the characterization of organic molecules, materials with supramolecular chirality, and in protein conformation determination, where there are distinctive spectral signatures for each secondary structure type, i.e., α-helices and β-sheets.^1^ Thus, CD spectroscopy is an important biophysical tool for characterizing native and modified proteins. In the biomedical context, protein misfolding can have dramatic consequences on cell physiology, causing serious neurodegenerative diseases, as found in Alzheimer's and Parkinson's diseases.^2^ Structural and kinetics studies of protein folding, using time-resolved approaches, are providing crucial insights at the molecular level into the etiology of these diseases.

There are two main ways to measure CD spectra: the ellipsometric method and the direct absorption difference detection method. The former is based on quantifying the variations of the ellipticity and azimuth orientation of a highly elliptically polarized beam passing through a dichroic sample.^3^ In 2012, Eom *et al.*^4^ innovatively adapted the ellipsometric method to a heterodyne-detection technique, providing both the CD spectrum and the optical rotation dispersion spectrum, by analysis of the phase and the amplitude of the transmitted orthogonal electric field of the incident light polarization. More recently, Hiramatsu *et al.*^5^ coupled the heterodyne detection technique to a singular value decomposition analysis. This improvement removes linear dichroism and linear birefringence artifacts, allowing accurate time-resolved CD (tr-CD) measurements in the visible range with subpicosecond temporal resolution. The second way to acquire a CD spectrum is based on absorption difference measurement. The light is alternately circularly right and left polarized using an optical or acousto-optic modulator. Then, the detection system records intensity variations and allows CD determination. This method has been extensively refined and can provide subpicosecond resolution for broadband measurements.^6^ Hache and colleagues used this technique to probe ultrafast kinetics in biomolecules^7^ and achieved 800 fs temporal resolution.^8^ Several groups^9,10^ have developed a setup for picosecond transient broadband CD measurements in the visible range. They used a combination of a Pockels cell and a light continuum generator to obtain a pulsed white light source alternately circularly right and left polarized. Recently, Preda and co-workers have introduced a new experimental arrangement based on the combination of time-domain detection and heterodyne amplification.^11^ This approach allows time-resolved optical activity measurements. Both ellipsometric and absorption difference broadband techniques have advantages and disadvantages, but they also often share the same spectral restriction to the visible range, notably due to the lack of stable continuum light sources in the far-UV range.

The synchrotron radiation circular dichroism (SRCD) technique was developed in 1980.^12,13^ Since its first use for protein structure determination,^14^ it has been used in a wide range of applications.^15–17^ Indeed, the brilliance of the synchrotron radiation (SR) in the vacuum UV (VUV) range and its stability enables one to measure CD spectra of samples dissolved in buffer down to 160 nm^18^ with an acceptable signal-to-noise ratio for the heterodyne detection.

In this paper, we describe the development of a different approach for SRCD measurements, utilizing the natural polarization of the SR emitted by a bending magnet.^19,20^ The DISCO beamline provides a SR beam composed of two parts (supplementary material, Fig. 1), which are above and below the electron's orbital plane in the synchrotron storage ring. These two continuum beams are similarly elliptically polarized with opposite directions (supplementary material 2). Schiller and Hormes^21^ have previously demonstrated that the natural elliptical polarization of the SR can be used for CD spectroscopy. However, they measured the CD signal sequentially, wavelength by wavelength; so the scanning process of the monochromator limited the temporal resolution of their setup significantly. We have developed a spectrograph that measures simultaneously the intensity of both (oppositely polarized) parts of the SR beam, allowing one to determine a whole UV CD spectrum in just a single measurement. While current SRCD setups only take advantage of the wide spectral range and the brilliance of SR, our setup also uses its natural polarization and its temporal distribution. This broadband single measurement approach gives access to a combination of a temporal and a spectral range previously inaccessible with the current broadband CD setups, enabling new insights into biomolecular dynamics.

**FIG. 1.**
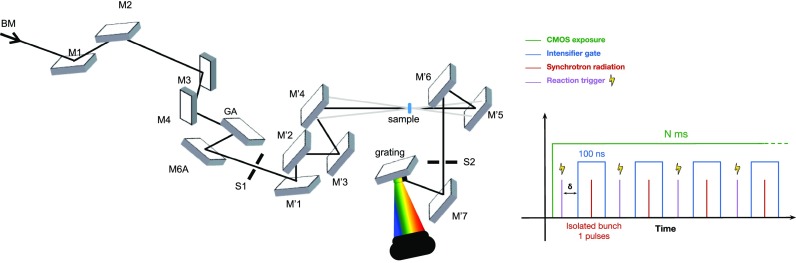
Left: Optical layout of the tr-SRCD setup with distances not at scale. Right: Detection sequence for single pulse measurement combined to a triggered system.

## METHOD

The tr-SRCD setup is annexed to the existing SRCD endstation at the DISCO beamline.^22^ The optical layout is shown in Fig. 1. The beamline excitation monochromator is used in grating first order for the wavelength calibration of the detection spectrograph (supplementary material, Fig. 3). With the zero order, we get on the sample a pulsed white beam containing all the wavelengths between 120 nm to 600 nm; this pulsed broadband source is used for the tr-SRCD measurement, limited by the CMOS spectral response. The beam is spatially defined by a spatial filter (labelled S1 in the figure) and then centered by the two-plane mirrors (M'1 and M'2) before being refocused on the sample. In order to avoid damage from beam irradiation, this optical system is designed to allow variation of the illuminated area on the sample. The spherical mirror M'3 and the cylindrical mirror M'4 refocused the beam 2975 mm after the monochromator slits; the calculated beam diameter at the focal plane is about 300 *μ*m (FWHM). By moving the sample cell by 200 mm along the optical axis, we get a controllable probe beam diameter from 300 *μ*m up to 3 mm. Adjustment of the beam diameter to the exposure time helps to minimize the sample irradiation dose. Beyond the sample, the cylindrical mirror M'5 and the spherical mirror M'6 horizontally focused the beam on a secondary slit (S2). This secondary slit at 3900 mm is used to define the final spectral resolution of the setup. The flat M'7 mirror reflected the beam on a spherical flat field grating with 580 grooves per mm (Horiba Jobin-Yvon) that diffract and focus the incident UV/vis light horizontally onto the 2D detector.

**FIG. 2.**
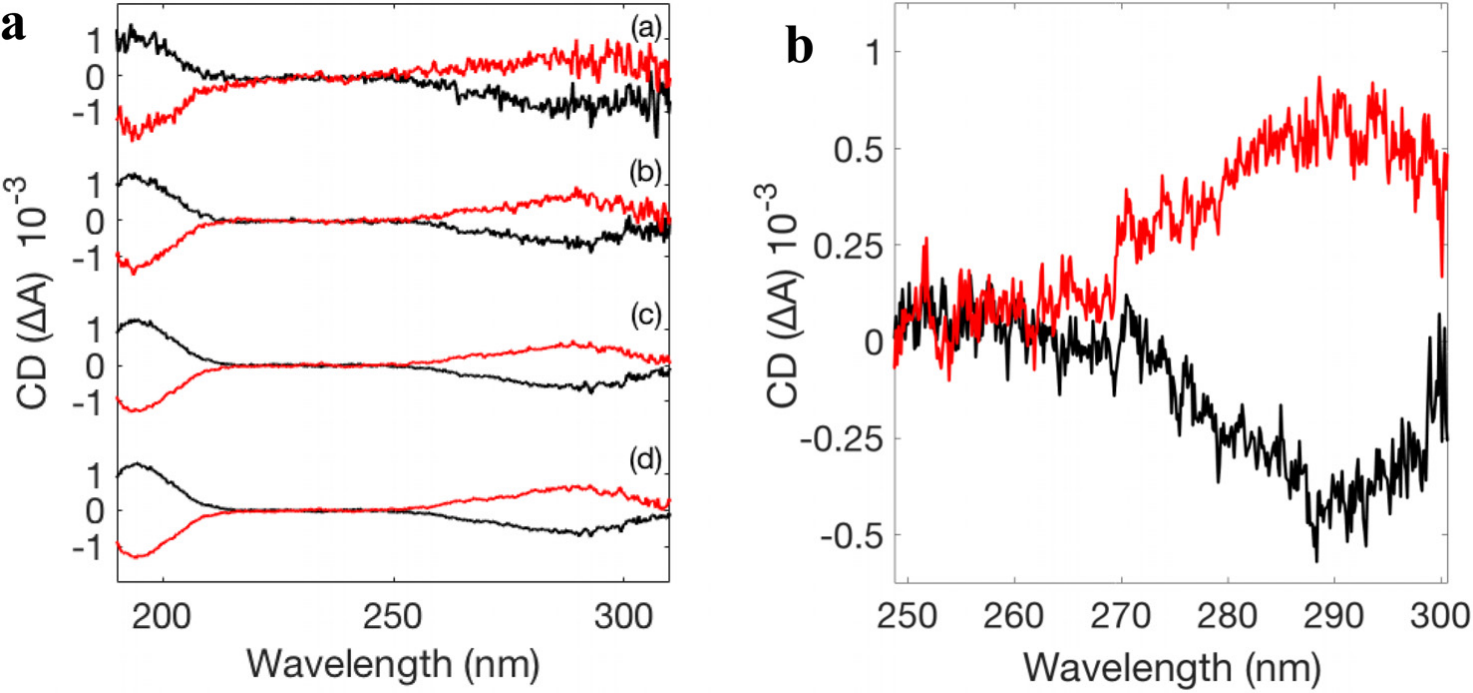
(a) CD spectra of D-CSA (black) and L-CSA (red) acquired with the tr-SRCD setup at 20 Hz with 500 *μ*s intensifier gate duration. 1, 10, 50, and 100 measurements were integrated to obtain the data shown in curves (a), (b), (c), and (d), respectively. (b) CD spectra of D-CSA (black) and L-CSA (red) from single pulse measurements acquired with the tr-SRCD setup at 500 Hz with 82 ps intensifier gate duration; 5000 measurements were integrated.

The tr-SRCD setup acquisition frequency is defined by the camera frame rate, which can be set from 0.033 Hz to 100 Hz in full resolution mode and can be increased further by reducing the pixel array size. This range of sampling frequency allows one to study reaction kinetics from tens of milliseconds to minutes through sequential measurements. The image intensifier can be synchronized with the camera output clock and with an external function generator, providing trigger signals with a higher frequency. When the intensifier is synchronized with the output clock, the temporal resolution of the measurement corresponds to the duration of the amplification gate or the light pulse length if the gate is sufficiently short (supplementary material, 4). However, if an external device triggers the image intensifier at a higher frequency than the camera frame rate, the exposure time of the camera defines the temporal resolution of the setup. The intensifier gate only amplifies the light coming from one SR pulse. The intensifier allows overcoming the limitation brought by the minimal exposure time of the CMOS (50 *μ*s) and so measures the intensity of one pulse at once (Fig. 1, right). We use this protocol for the results presented in Fig. 2(b). We integrated 500 pulses for each recorded image for this measurement.

The sample was held in a CaF_2_ flow cell with a path length of 24 *μ*m (Aquaspec, Microbiolytics GmbH, Freiburg, D). The sensitivity ranges of 2D detectors, such as CDD or CMOS, are usually limited to 200 nm. We may also use an image intensifier (HS–IRO, LAVISION) to reach the lower wavelength range and to increase time resolution. To recover the main part of the visible light provided by the HS-IRO phosphor, a combination of two 50 mm focal length infinity corrected objectives was used. A CMOS detector (Prime 95-B, PHOTOMETRICS) records the phosphor screen image. For the applications that do not need the picosecond temporal resolution, the Prime 95-B was used without intensifier.

Equation (1) defines CD at a given wavelength. The variables *A_L_* and *A_R_* correspond to the absorption for left and right-handed circularly polarized light, respectively. All the spectra associated with the tr-SRCD setup in this study were determined by the following equation:

$$CD=(A_{L}-A_{R}).$$ (1)

In this case, the absorption difference between left-handed and right-handed circularly polarized light can be associated with the absorption difference between the upper and lower part of the SR (supplementary material, Note 5). These portions of the image are selected in order to show opposite polarizations. Although such procedure reduces the photon flux at each wavelength, the masks applied remove at most 25% of the images intensity, giving a moderate effect on the photon flux useful for the measurements.

## RESULTS

We chose camphorsulfonic acid (CSA), purchased from Sigma-Aldrich, as our calibration sample, as it has two strong peaks in the VUV/deep UV range: the first at 290 nm and the second at 192 nm. It is commonly used as a calibration standard for CD spectrometers.^23,24^ To validate that the recorded signal is CD, the two enantiomers must exhibit opposite signed spectra.

The resulting spectra for D-CSA and L-CSA, measured between 190 nm and 315 nm for the 30 mg/mL solution concentration, are shown in Fig. 2(a). The CMOS detector has been used without the image intensifier. It integrated the light for 500 *μ*s, and the images were acquired at 20 Hz. As expected, we observe two oppositely signed spectra with identical amplitudes. The ratio between the amplitude of the two peaks should be about 2;^24^ we obtained 2.11 +/– 0.11 in the tr-SRCD spectra [curve (d) of Fig. 2(a)]. The signal-to-noise ratios at 190 nm for the D-CSA spectra (a), (b), (c), and (d) in Fig. 2(a) are 6.6, 12.7, 39.8, and 44.40, respectively.

In order to establish the highest theoretical temporal resolution of the setup that is attainable experimentally, we added an image intensifier, thereby obviating the temporal resolution limitation of the CMOS detector. We performed this steady state experiment on the CSA with the maximal temporal resolution as a proof-of-principle. The intensifier was triggered with an external trigger, and its amplification was reduced to its shortest value, 100 ns. The measurements were made using the 8-bunch mode of the synchrotron SOLEIL which provides 82 ps pulses at 7.14 MHz. The intensifier amplified only one pulse per gate. So its temporal resolution corresponds to the pulse length, which, in this case, is 82 ps. In Fig. 2(b), each spectrum comes from ten images; 500 separated pulses were integrated for each image. The spectral range was reduced to between 255 nm and 300 nm in order to optimize the setup for the detection of the peak at 290 nm. The signal-to-noise ratio at 290 nm for the D-CSA spectrum is 10.33.

**FIG. 3.**
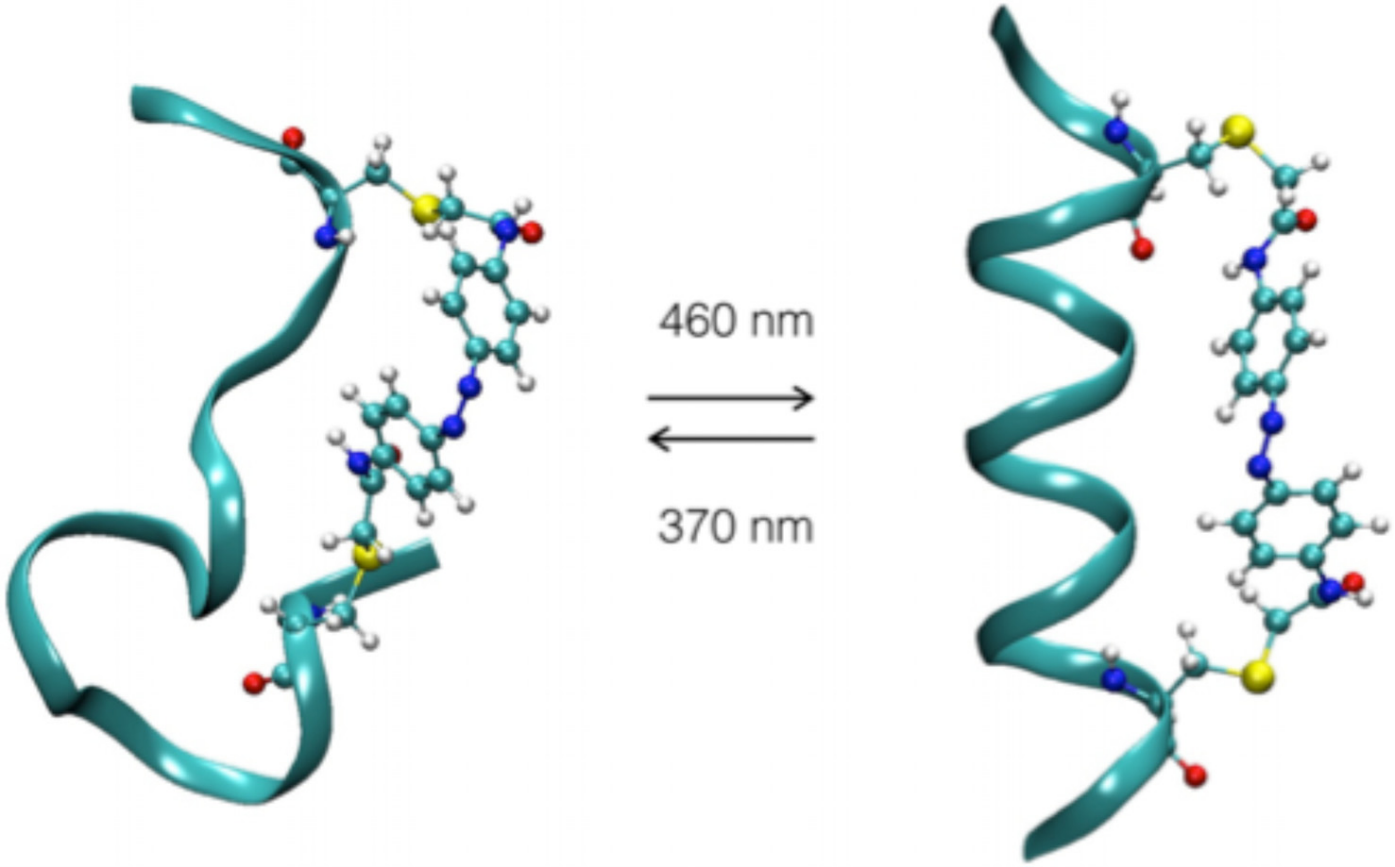
Schematic representation of the FK-11-X molecular system for both *cis* and *trans* conformation. Its amino acid sequence is Ac-Glu-Ala-Cys^AZO^-Ala-Arg-Glu-Ala-Ala-Ala-Arg-Glu-Ala-Ala-Cys^AZO^-Arg-Gln-NH_2_.

**FIG. 4.**
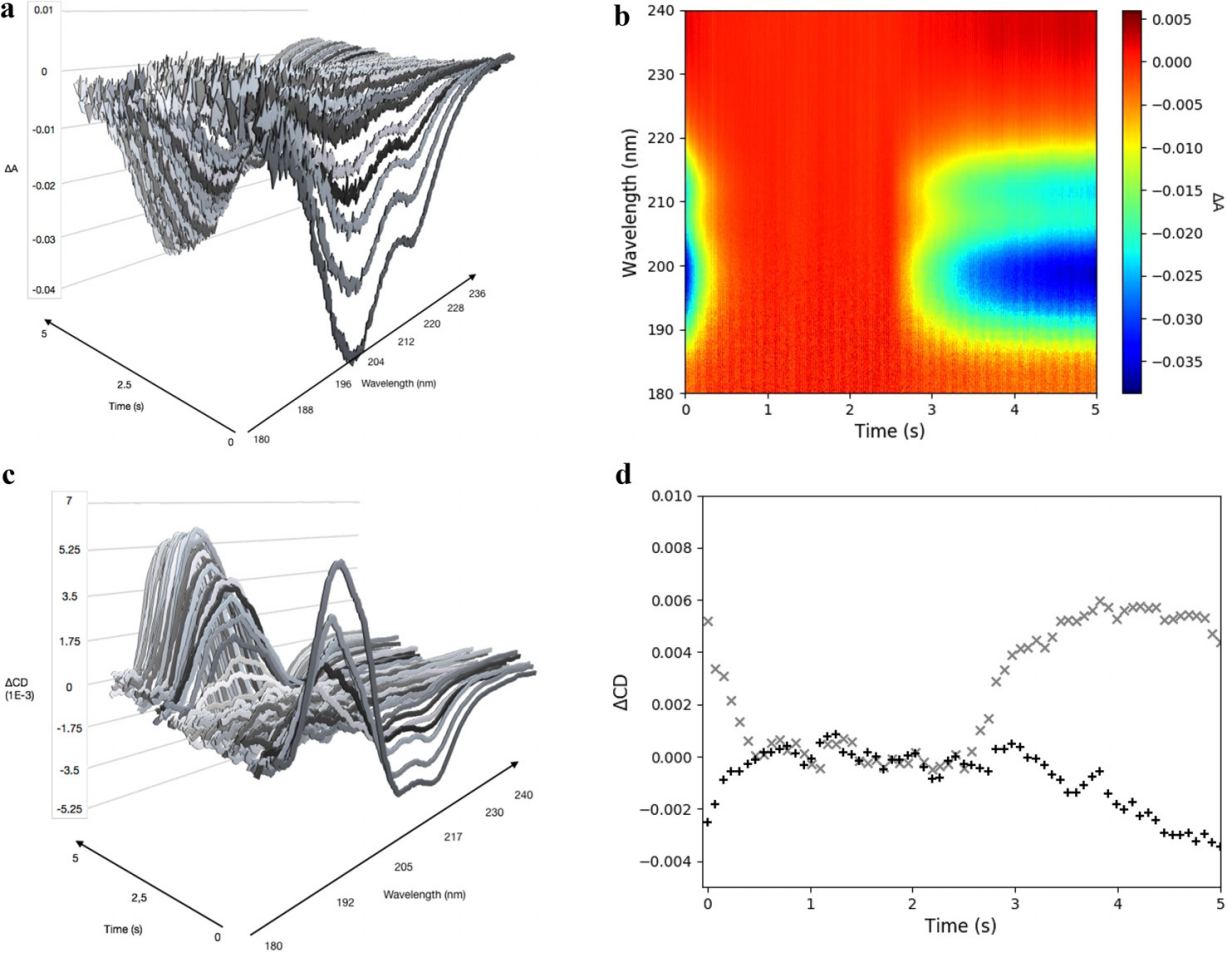
FK-11-X evolution from *trans* to *cis* over time after 460 nm triggering and relaxation after 370 nm triggering. (a) Evolution of the absorbance difference during the 370 nm irradiation. One spectrum every 70 ms is represented. (b) 2D representation of the evolution of the global absorbance over the cycle; 650 spectra are shown. (c) Evolution of the CD difference during the 370 nm irradiation. One spectrum every 70 ms is represented. (d) Evolution of the CD at 190 nm (x) and 204 nm (+) over the cycle.

We tested the capacity of the setup to follow reactions in real time by studying a photocontrolled and reversible system, the azobenzene crosslinked peptide FK-11-X.^25,26^ It is composed of a 16 amino acid peptide cross-linked to a photoswitchable molecule: azobenzene (Fig. 3). This ligand can be isomerised from the *trans* to *cis* and *cis* to *trans* conformation using a 370 nm and a visible light source (460 nm), respectively. Several groups have studied the dynamics of this photo-induced reaction, and the isomerization of the azobenzene appears to occur within one picosecond.^27–30^ This conformational change constrains the peptide structure and triggers its folding and unfolding processes. Theoretical^25,31^ and experimental^32,33^ studies agree that, following the azobenzene isomerisation, peptide conformational changes occur on the microsecond scale. The azobenzene cross-linked peptide was synthesized as described previously^25^ (supplementary material, Note 6). The aim of our study was to follow the change in the concentration of the unfolded (*cis* azobenzene) and folded (*trans* azobenzene, ground state) FK-11-X peptide. We triggered the isomerization with two continuous diodes, at 370 nm (5 W) and at 460 nm (5 W). The concentration of FK-11-X solution was 10 mg/ml in phosphate buffer (70 mM, pH 7). The sample was alternately irradiated with the two diodes. The sample was first irradiated 2.5 s with the 370 nm diode to trigger the *trans* to *cis* isomerisation and then 2.5 s with the 460 nm diode to trigger the *cis* to *trans* isomerization. The transient CD and absorption spectra of isomer concentration changes were followed, measuring spectra at 130 Hz with a 520 *μ*s temporal resolution; the measurement has been integrated over 39 cycles of alternate irradiations. The variations in the absorption and CD are shown in Fig. 4.

The isomer concentration change induces a variation in the absorption of the sample. The evolution of the absorption measured by the detector is shown in Figs. 4(a) and 4(b). The absorption variation [Fig. 4(a)] shows two peaks, at 198 nm and 212 nm. It decreases progressively and reaches a plateau where ΔA ≃ 0 in the whole range of interest because the intensity reference is measured from these time frames. A clear decrease in intensity occurs from the first milliseconds after the 370 nm irradiation. The irradiation at 460 nm that triggers the *cis* to *trans* isomerization starts at 2.5 s. The measured intensity increases progressively over the following 2.5 s to reach the initial value measured at t0. This plot shows that the *trans* to *cis* isomerization is completed for the main part of the molecules after 1 s of irradiation with the 370 nm diode. This plateau does not correspond to an equilibrium between the diode flux and the lifetime of the *cis*-isomer. Indeed, the FK-11-X peptide is relatively stable in its *cis* configuration, and it needs tens of minutes to naturally switch from the *cis* to the *trans* configuration. It also shows the complete reversibility of the concentration ratio. The intensity reference *I_0_* for the determination of the absorbance variation and so for the CD calculation corresponds to the average of the intensity measured between 1 and 2.5 s. In this time range, almost all the molecules are in their *cis* configuration. Thus, the absorbance and CD variation measurements shown in Fig. 4 correspond to the variation from the absorbance and the CD spectrum of a solution of *cis* FK-11-X peptides. The CD variation spectra [Figs. 4(c) and S5] are close to the signal of the α-helical structure, and the increasing amplitude reflects the change of the species concentration. The first spectrum corresponds to the total difference between the CD spectrum of the unfolded (*cis*) FK-11-X peptide and its folded (*trans*) configuration. Then, the concentration of the *trans* configuration decreases progressively during the 370 nm irradiation and so the amplitude of the CD difference decreases as well until it reaches approximately 0 before the first second of irradiation. The CD variation at 204 nm and 190 nm [Fig. 4(d)] also confirms the reversibility of the system, and the CD difference returns to its initial value at t0 after the 2.5 s of irradiation with the 460 nm diode. The total CD changes from the initial state and the final state measured with the tr-SRCD setup (Fig. S5) and from steady state measurement (*cis* and *trans* CD spectra) in the literature^32^ are similar. This comparison shows that being able to calibrate the SR polarization, we can measure transient CD spectra similar to those measured with steady state setups. The only difference arising from the elliptical polarization is the amplitude of the CD, which is linearly correlated with the circularity of the polarization.

Our study demonstrates that the new tr-SRCD setup developed at the DISCO beamline^22^ of the SOLEIL synchrotron radiation facility offers new opportunities for the investigation of biomolecular structure and dynamics. The setup is based on the use of the natural properties of the synchrotron radiation provided by the DISCO beamline. It uses both spectral and polarization characteristics to reduce the time required for the measurement of a transient CD UV spectrum. Our tr-SRCD measurements of the CSA show that an integration time of 500 *μ*s is enough to obtain an acceptable signal-to-noise ratio, i.e., greater than 6. We performed a proof-of-principle high temporal resolution steady state experiment on CSA performed using an image intensifier allowing resolution down to a few tens of picoseconds. The FK11 experiment demonstrates the capacity of the tr-SRCD setup to follow dynamics in real time, with a temporal resolution of 500 *μ*s and acquired at 130 Hz. These values do not correspond to the hardware cap and can be easily improved by opening the spectrograph slits and integrating more photons in a shorter time period and/or coupling the system with the image intensifier. This breakthrough in the SRCD spectroscopy will enable the study of many biological and chemical reactions crucial for our understanding of biomolecular phenomena.

The development of new CaF2 microlenses windows for sCMOS camera would grant access to direct detection and measurement of spectra on the full DUV range down to 160 nm.

In the near future, the setup could easily be coupled to other reaction triggers, such as a T-jump, lasers, and microfluidic stop-flow equipment. Thus, this new approach will provide exciting insights into the dynamics of biomolecules, as well as for molecular and materials systems more broadly. This method will enable one to follow the behavior of molecules through high quality SRCD spectra on a temporal range from the picoseconds to minutes. Indeed, it covers time scales consistent with the fluctuation and domain motions of proteins suspected to play a fundamental role in their activity. Ongoing experiments involve the kinetic study of the refolding of model proteins after thermal denaturation and the following of DNA nanostructure formation.^34^

## SUPPLEMENTARY MATERIAL

See the supplementary material for the details on (1) the beam, (2) the polarization characterization, (3) the wavelength calibration, (4) the detection scheme with an intensifier, (5) the detailed algorithm, and (6) the chemical steps of purification.
